# 675. Crimean-Congo Hemorrhagic Fever Beyond Ribavirin: A Systematic Review

**DOI:** 10.1093/ofid/ofab466.872

**Published:** 2021-12-04

**Authors:** Stephanie P Fabara, Raghavendra Tirupathi, Juan Fernando Ortiz, Urvish Patel, Sashwath Srikanth, Jaffar A Al-Tawfiq, Ali A Rabaan

**Affiliations:** 1 Universidad Catolica De Santiago De Guayaquil, Guayaquil, Guayas, Ecuador; 2 WellSpan Health, Chambersburg, Pennsylvania; 3 Larkin Community Hospital, Miami, Florida; 4 Mount Sinal Medical Center, New York, New York; 5 Patient First Medical Clinic, San Diego, California; 6 Johns Hopkins School of Medicine, Dhahran, Al Bahah, Saudi Arabia; 7 Johns Hopkins Aramco Health Care, Dhahran, Al Bahah, Saudi Arabia

## Abstract

**Background:**

The Crimean-Congo Hemorrhagic Fever (CCHF) is a tick-borne virus infection that has been reported in about 30 countries worldwide. Clinical presentation is divided into three phases: pre-hemorrhagic, hemorrhagic, and convalescence. Ribavirin is standard of care treatment for acute infection and prophylaxis. However, the use of other treatments beyond ribavirin is largely unknown.

**Methods:**

We conducted a systematic review using MOOSE protocol. The inclusion and exclusion criteria are seen in the Prisma diagram. For Bias Analysis we use a Robin-1 tool.

Literature review algorithm

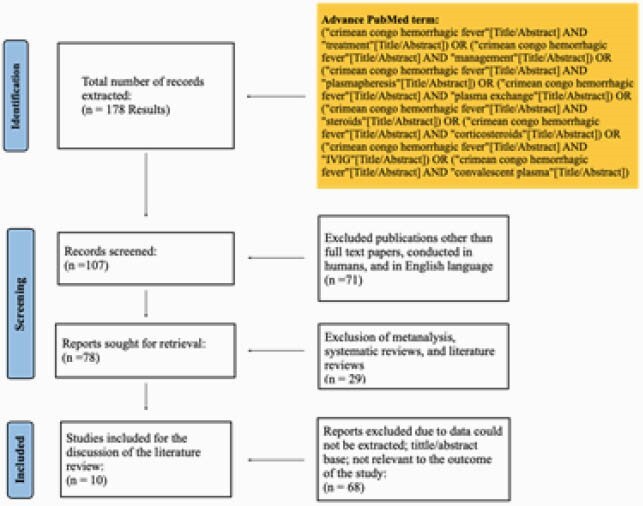

**Results:**

We gathered a total of 10 studies, which included 4 therapeutic plasma exchange (TPE), 2 corticosteroids, 2 IVIG, and 1 with convalescent plasma (CP).

TPE in one study showed decreased mortality rate and increased efficacy in patients with severe CCHF. While the other study reported pulmonary embolism related to the use of TPE. Nevertheless, the patients had good outcome in the end. Two case reports used TPE plus ribavirin and supportive measures. Both were discharged home and recovered without sequela. Corticosteroids were found to be beneficial in one study were the case fatality rate was lower with the addition of corticosteroids to ribavirin in severely ill patients (p=0.0014). In a case series of six patients, who received the combination in early stages of the disease had good clinical outcomes with improved survival. IVIG was shown to increase platelet counts in two studies. In the first study, platelet count increased above 150,000/mL in 8.5 +/- 2.5 days. While in the other study the normalization of platelets was seen in 4 - 4.8 days, with no significant difference (P = 0.49). In addition, there was a decrease in the duration of symptoms but there was no statistically significant difference in mortality rates (P = 0.171). CP treatment showed a survival rate of 86% in treated patients. CP was more useful in high-risk patients, defined as having a viral load of 10^8^ copies/mL or more. The main limitations of the studies were the sample size and heterogenicity among the outcomes of the studies.

**Conclusion:**

TPE, CP, IVIG, and corticosteroids were effective in improving the clinical outcomes of the patients. The use of these treatments beyond ribavirin should be explored in future studies.

**Disclosures:**

**All Authors**: No reported disclosures

